# Antimalarial drug discovery: targeting the hypnozoite for new radical curative agent

**DOI:** 10.1186/1475-2875-11-S1-O32

**Published:** 2012-10-15

**Authors:** Bryan Yeung

**Affiliations:** 1 Novartis Institute for Tropical Diseases, Singapore

## 

*Plasmodium vivax* malaria remains a significant health burden in endemic areas like South East Asia, Central and South America. *P. vivax* infections are characterized by relapses of malaria caused by persistent liver stages of the parasite (hypnozoites) which are not present in *P. falciparum* infections. Currently, the only approved treatment option for the radical cure of P. *vivax* malaria is the 8-aminoquinoline, primaquine. However the long treatment course (two weeks) and severe side effects (hemolytic anemia in G6PD-deficeint patients) highlights the need for new chemical classes of drugs.

Currently the discovery of new anti-hypnozoite drugs is limited to testing in the *P. cynomolgi* rhesus monkey model. However recently developed assays that allow for the testing of new chemical entities on the parasite liver-stages (schizonts) and sexual stages (gametocytes) could be leveraged to identify dual acting compounds with potental anti-hypnozoite activity. In addition, compounds which target more than one stage in the Plasmodium lifecycle would be highly valued and compliment the current arsenal of antimalarial drugs.

